# Rural Zulu women's knowledge of and attitudes towards medical male circumcision

**DOI:** 10.4102/phcfm.v7i1.775

**Published:** 2015-03-31

**Authors:** Joseph N. Ikwegbue, Andrew Ross, Harbor Ogbonnaya

**Affiliations:** 1 Department of Family Medicine, University of KwaZulu-Natal, South Africa

## Abstract

**Background:**

Medical male circumcision (MMC) is a key strategy in the South African HIV infection prevention package. Women may have a potentially powerful role in supporting such a strategy. Circumcision is not a traditional part of Zulu society, and Zulu women may have limited knowledge and ambivalent or negative attitudes towards MMC.

**Aim:**

This study employs quantitative data to expand insight into rural Zulu women's knowledge of and attitudes towards MMC, and is important as women could potentially yield a powerful positive or negative influence over the decisions of their partners and sons.

**Setting:**

A hospital-based antenatal clinic in rural KwaZulu-Natal.

**Methods:**

Participants were 590 pregnant, mostly isiZulu-speaking women. Data on their knowledge of and attitude towards MMC were collected using a questionnaire and were analysed descriptively.

**Results:**

The majority of the women supported MMC; however, knowledge of the potential benefits was generally poor. Most would encourage their partners and sons to undergo MMC. The preferred place for the procedure was a hospital.

**Conclusion:**

Zulu participants supported MMC and would support their partners and children being circumcised. Knowledge around potential benefits was worryingly poor, and further research into disseminating information is essential. The findings highlight the need for an expanded campaign of health education for women, and innovative means are suggested to enhance information accessibility. Reasons for preferring that MMC be carried out in hospital need to be explored further.

## Abstrait

**Connaissances et attitudes des femmes zouloues de la campagne à l’égard de la circoncision médicale masculine.**

**Contexte:**

La circoncision médicale masculine (MMC) est une stratégie clé du programme sud-africain de prévention du VIH. Les femmes ont un rôle potentiellement important de soutien dans cette stratégie. La circoncision n'est pas une tradition zouloue et les femmes zouloues ont une connaissance limitée et des attitudes ambivalentes ou négatives envers la MMC.

**Méthode:**

Les 590 participantes étaient des femmes enceintes, en majorité d'expression isiZulu qui fréquentaient une clinique prénatale d'un hôpital de campagne au KwaZulu-Natal. Les données sur leurs connaissances et leur attitude envers la MMC ont été collectées au moyen d'un questionnaire et analysées de façon descriptive.

**Résultats:**

La majorité était en faveur de la MMC, mais elles avaient peu de connaissances des avantages potentiels de la procédure. La plupart voulaient encourager leur partenaire et leurs fils à subir la MMC. L'hôpital était l'endroit préféré pour subir l'opération.

**Discussion:**

Les résultats montrent fortement le besoin de faire une campagne élargie d’éducation sanitaire pour les femmes et des moyens innovants sont proposés pour améliorer l'accessibilité de l'information. Il faudra explorer d'avantage pour quelles raisons elles préfèrent que la MMC soit faite dans les hôpitaux.

**Conclusion:**

Les participantes zouloues sont pour la MMC et encourageraient leur partenaire et leurs fils à être circoncis. Les avantages potentiels de la procédure étaient peu connus et il faudra absolument chercher à diffuser des informations.

## Introduction

In 2007 the World Health Organization (WHO) estimated that 33 million people worldwide were HIV positive and that 35% lived in sub-Saharan Africa.^1^ In South Africa a Department of Health report in 2014 estimated that 5.7 million South Africans were HIV positive, representing 12% of the South African population.^2^ Numerous international studies indicate that medical male circumcision (MMC) can significantly reduce the incidence of HIV infection in males.^3^ Local South African studies report a 60% reduction in risk of HIV infection in men who have been circumcised.^4^

In 2007 the WHO and UNAIDS recommended that MMC should be a priority HIV prevention intervention in countries with a high HIV infection prevalence and low prevalence of male circumcision.^5^ As South Africa meets these criteria, the South African National Department of Health adopted MMC as a key preventative initiative. In 2010 a campaign was initiated to roll out MMC, aiming to circumcise 5.7 million or 80% of men aged 15–49 years over a 5-year period.^6^ Historically male circumcision is already a cultural practice amongst sectors of the mainly rural population of South Africa, including in the Xhosa-, Venda- and Sotho-speaking ethnic groups.^7^ However, literature reports that circumcision is not part of Zulu cultural traditional practices.^8,9^ Although the practice of male circumcision was supported by the Zulu king in 2010, the practice is still considered somewhat alien amongst the isiZulu-speaking population.^9^ As a consequence Zulu men may be potentially reluctant to undergo MMC as it is not part of their culture.

Willingness to undergo MMC may also be influenced by knowledge of the procedure and its potential benefits and challenges. A qualitative study in rural KwaZulu-Natal in 2012 reported that men generally did have adequate knowledge of the potential benefits of MMC and expressed willingness to undergo the procedure.^10^ Interestingly, the study reported that ‘men's positive attitude could be enhanced by women's endorsement and increased participation in MMC promotion’.^10^

It is encouraging that women could play a potentially positive role in supporting the MMC campaign. However, as circumcision is culturally unfamiliar, Zulu women may have an ambivalent or somewhat negative attitude towards supporting men in carrying out MMC. There may also be some ambiguity or negativity around MCC for other reasons, including that it may be associated with a negative effect on sexual pleasure.^11,12^ Rural women's knowledge around potential benefits and challenges of MMC may be lacking, as they may have poor access to health information in general and about MMC in particular. A large multi-centre international study found that although women generally support MMC programmes, they often lack factual knowledge about the benefits and risks of MMC and its role in HIV infection prevention.^13^ A study of information availability and utilisation by rural women in KwaZulu-Natal reported a need for suitable media where information on issues such as health, agriculture, education, business and legal matters could be made more readily available.^14^

The majority of studies on MMC have comprised male participants and there is correspondingly less literature available on the knowledge and opinions of women about MMC. A meta-analysis of 13 studies indicated that only four included women participants.^16^ A small study in rural KwaZulu-Natal included 44 women and reported that 68% of the sample was in favour of male circumcision, and that 73% would circumcise their young sons.^10^

The main predictors of support of circumcision revolved around the women's knowledge about the relationship between male circumcision status and reduction of acquisition of sexually transmitted infections, including HIV; a higher knowledge was associated with an increased likelihood of MMC support. The greatest logistical barrier to MMC that was identified was that circumcision could only be carried out by trained hospital doctors.^15^ This restraint may be particularly pertinent in rural areas, as hospitals may not be readily accessible.

It cannot be assumed that rural women have knowledge of circumcision; for example, a knowledge, attitude and practice study in South Africa revealed that one-third of the female participants could not identify a circumcised penis.^17^ It is also of concern that 3% of participants in this study thought that circumcised men were fully protected against HIV; as a result of such misconceptions, women may not request their partners to use condoms during sexual intercourse.

It is pertinent to expand the literature around rural Zulu women's knowledge of MMC for several reasons, including the following: they may not know of the potential benefits; their cultural norms may mitigate against it; they may feel that MMC would have a negative effect on, for example. sexual pleasure. Rural women may be particularly naive about the potential benefits of MMC as they may have poor access to technology, the media and health information. They may be less willing than their urban counterparts to support MMC as hospitals may be far from their homes and access may be logistically and financially challenging.

A literature review indicates that there has been a qualitative review of the knowledge and attitudes of women around MMC in rural KwaZulu-Natal.^10^ This current study employs quantitative data to expand the knowledge, and is important as women could potentially yield a powerful positive or negative influence over the decisions of their partners and sons.

## Research methods and design

### Study design

This was an exploratory study; such studies are carried in areas of research where there is little existing information.^18^ The study was observational, descriptive and cross-sectional and ran from April to August 2012.

### Setting

The study site was an antenatal clinic at the regional obstetric hospital in rural northern KwaZulu-Natal. The site was specifically selected as the study intended to assess the knowledge and attitudes of rural women.

### Study population and sampling strategy

The study population was mostly isiZulu-speaking African women attending the antenatal clinic over the study period. This population was purposively selected as being potentially information-rich key informants.^18^ It is particularly important to study these women as they may be having unprotected intercourse and are at risk of HIV infection. On average 1000 women attend the antenatal clinic at the study site every month. A sample size of 600 women, representing 60% of the study population, was considered to be sufficient for this exploratory study (exploratory studies typically sample 30% of the study population).^18^ All women attending the antenatal clinic were invited by the clinic nurse to partake in the study. During routine antenatal health education sessions, the purpose of the study was explained by the nurse to the women. Potential pressure to join the study was thus minimised, as the nurse was not a member of the research team. Women were presented with a Study Information Sheet and Consent Form, both of which were available in English and isiZulu. Women were invited to participate until the required sample size was reached.

### Data collection

Data were collected using a structured questionnaire which was based on the findings of a qualitative study carried out a nearby study site.^10^ This qualitative review involved 44 women and data were collected using focus group discussion. The themes arising from this study were summarised as a questionnaire, and initial content analysis by members of the research team and nurses at the study site indicated that potential participants would be able to understand it and respond to the questions. The questionnaire included information on demographic features (age, marital status, education level). Questions generated from the earlier qualitative study considered the following:

- Do you feel that MMC protects men against HIV?
- Would you support your partner to go for MCC?
- Do you feel that MMC improves sexual satisfaction?
- Does MMC protect your partner from HIV?
- Would you support MMC for a cultural reason?
- Would you circumcise a male child?
- Where would you prefer MMC to be held?
- Do you think MMC should be part of HIV prevention?
- Do you feel that MMC increases male promiscuity?
- Do you feel that MMC will cause sexual problems?

The questionnaire was available in both English and isiZulu and was distributed to participants who consented to participate in the study. A research assistant and nurse were available to assist women if they reported difficulty in understanding the questions. A box was left in the antenatal clinic where completed questionnaires were dropped off and later collected by a research assistant. A total of 590 questionnaires were returned out of the 620 distributed.

### Data analysis

Data were entered into Excel and analysed descriptively using SPSS version 21.

## Ethical considerations

Ethical permission for the study was given by the Biomedical Research Ethics Committee of the University of KwaZulu-Natal (BE136/11), the study site (hospital) ethics committee and the Provincial Department of Health.

## Results

The results are presented as follows: (a) demographic features; and (b) knowledge and attitudes.

### Demographic features

The majority of the participants were in the age range 20–39 years (86%), 15.7% were married, a quarter (23.2%) had only primary school education, and half (53%) had completed matric. In terms of ethnic background, 98% considered themselves to be Zulus, with two Xhosas and two Sothos. This and other demographic information is presented in Table 1a and Table 1b.

**TABLE 1a:** Demographic profile of participants.

Age group (yrs)	Frequency (%)	Educational status	Frequency (%)
10–19	65 (11.3%)	University/higher institution	111 (19.3%)
20–29	337 (58.7%)	Matric	313 (53.1%)
30–39	157 (27.4 %)	Primary	137 (23.6%)
40–49	15 (2.6%)	None	15 (2.6%)
Missing data	16	-	15
**Total**	**574**	**-**	**575**

**TABLE 1b:** Demographic profile of participants.

Marital status	Frequency (%)	Employment status	Frequency (%)
Married	90 (15.7%)	Employed	90 (15.3%)
Not married	484 (84.3%)	Unemployed	500 (84.7%)
Missing data	16	-	-
**Total**	**574**	**-**	**590**

### Knowledge and attitudes

Table 2 summarises aspects of knowledge and attitudes of MMC amongst women who participated in the study. Two-thirds (64%) reported that they knew the meaning of MMC. Only half (47.9%) felt that MMC could protect men against HIV infection. Interestingly, despite this reported low level of knowledge about the potential benefits, most (82.4%) responded that they would encourage their partner to go for MCC. Most did not relate MMC to improvements in sexual satisfaction (91.5%). Two-thirds (66%) were supportive of the MMC programme to protect their partners from HIV infection. Most did not support MMC as a cultural practice (90%). Encouragingly, most (83.7%) would take their son for circumcision. The preferred place for circumcision was a hospital (86.5%). It was of concern that 90% felt that MMC may increase male promiscuity. The Pearson Chi-square test showed a significant association between level of education and the meaning of male circumcision (*p* = 0.009) and an inverse association between level of education and understanding that MMC protects against HIV (*p* = 0.004).

**TABLE 2:** Knowledge and attitudes.

MCC amongst woman	Answer	Frequency
Knows the meaning of MMC	Yes	380 (64.4)
	No	210 (35.5)
**Total**		**590**
Supports MMC to protect partner from		
HIV infection	Yes	391 (66.3)
	No	199 (33.7)
**Total**		**590**
Feels that MMC protects men		
against HIV infection	Yes	271 (47.9)
	No	64 (11.3)
	Not sure	231 (40.8)
	Missing data	24
**Total**		**566**
Supports MMC for cultural reasons	Yes	55 (9.3)
	No	535 (90.7)
**Total**		**590**
Supports partner going for MMC	Yes	459 (82.4)
	No	25 (4.5)
	Not sure	73 (13.1)
	Missing data	33
**Total**		**557**
Effect of MMC on sexual pleasure	Increase	158 (29)
	Decrease	45 (8.3)
	Not sure	341 (62.7)
	Missing data	46
**Total**		**544**
Believes MMC will improve		
sexual satisfaction	Yes	50 (8.5)
	No	540 (91.5)
**Total**		**590**
Would circumcise male child	Yes	471 (83.7)
	No	36 (6.4)
	Not sure	56 (9.9)
	Missing data	27
**Total**		**563**
Preferred place for circumcision	Hospital	480 (86.5)
	Surgery	45 (8.1)
	Traditional	20 (3.6)
	Others	10 (1.8)
	Missing data	35
**Total**		**555**
Supports MMC being integrated		
into HIV prevention package	Yes	418 (70.8)
	No	18 (3.1)
	Not sure	103 (19.1)
	Others	-
	Missing data	51
**Total**		**539**
Feels MMC will increase male		
promiscuity	Yes	56 (9.5)
	No	534 (90.5)
**Total**		**590**
Feels MMC will cause sexual problems	Yes	5 (0.8)
	No	585 (99.2)
**Total**		**590**

MMC, Medical male circumcision.

## Discussion

The demographic profile of the participants is similar to that of other studies on antenatal clinic attendance at district hospitals in South Africa, with the majority of women in the 20–29 years age group (58.7%) and 11% in the 10–19 years age group.^19^ Most of the women in this study were unemployed and unmarried. It is of concern to note that 24% (137/575) of the women that responded had only primary education and that only 19% (111/575) had exposure to higher education. Although education is seen as a priority by many rural communities, a report by the Global Movement for Children^20^ highlighted the barriers to female education in many rural communities, including the preference for male education in most families. This finding is significant, as a meta-analysis sponsored by the World Bank suggested that female education increases women's negotiating power, narrows the inequality between men and women and improves the overall health status of women, including their ability to negotiate condom use.^21^

It is reassuring that participants reported a high level of support for circumcision, and this finding is consistent with other local studies which have shown MMC to be an acceptable preventative intervention amongst female pharmacy and nursing students.^20^ A high level of women's support for MMC is reflected in other international studies.^16^

Participants displayed a poor knowledge of the role of MMC in prevention of HIV infection transmission, with only half of the participants aware that MMC is important for prevention of HIV infection. This lack of knowledge is consistent with the findings of other studies.^23^ Analysis showed a positive association between level of education and the reported knowledge of the meaning of male circumcision (*p* = 0.009). It was somewhat surprising that a higher level of education had an inverse relationship with the knowledge that male circumcision protects against HIV infection (*p* = 0.004). Further studies are needed to review issues around this unexpected finding.

These findings highlight the need for expanded dissemination of information about the benefits of MMC to women. There is an urgent need to employ strategies to enhance women's access to knowledge around MMC. Existing literature also stresses the importance of finding suitable media for dissemination of health information to rural women.^14,24^ In rural KwaZulu-Natal there is a unique intervention around increasing information accessibility found in the Mpilonhle Project. This project makes use of mobile health and education units in rural schools and communities in KwaZulu-Natal to provide medical and social services, give HIV counselling and education, and assist people to improve their computer skills.^25^ Such existing projects could be sourced and encouraged to introduce information-sharing for women around MMC in the range of interventions available.

It was encouraging that despite a lack of knowledge about the benefits of MMC most participants were supportive of the MMC programme and would encourage their partners and sons to be circumcised. The high level of support for childhood male circumcision is similar to the findings of studies carried out elsewhere.^26^ This important finding must be further investigated, as studies suggest that infant circumcision is also an effective strategy for prevention of transmission of HIV infection and other sexually transmitted infections.^28^

A positive finding was that a minority thought that MMC would reduce sexual pleasure and that most did not expect MMC to cause any sexual problems nor lead to greater promiscuity amongst men. These are reassuring findings, because if circumcision was perceived to reduce sexual pleasure, cause sexual dysfunction or lead to greater promiscuity, women's support for the roll out of MMC may be lessened.

The preferred place for the MMC procedure was in a hospital, and this is significant since a rural population may have poor access to a hospital facility. That there was a preference for hospitals may reflect that circumcision is not routinely carried out as a traditional practice in this rural Zulu community. It would be interesting to compare this finding to that amongst potential Xhosa participants in the Eastern Cape where circumcisions are commonly carried out in the community. However, Xhosa women may also support circumcision in a hospital due to the high mortality and morbidity associated with community-based circumcision.^25,26,27^

The majority of respondents supported the integration of MMC into an HIV prevention package, and this finding is consistent with those of other studies, and may reflect the support for any programme aimed at preventing HIV and/or AIDS.^29,30^

### Limitations

A limitation in the study lay in the data collection method. Questions only assessed whether women thought they knew about MMC, and an actual knowledge assessment did not occur. Further studies on actual knowledge and not reported knowledge are required. The views of those who did not agree to participate were not explored, and this may be significant as women who have particularly strong negative views of circumcision may have refused to participate.

## Conclusion

It was reassuring that women supported MMC and would advocate it. The low level of knowledge of the potential benefits of MMC was concerning and points towards a need for expansion of information accessibility for these women. The preference for hospital-based MMC may limit access, and further study is required to assess whether this finding is particular to this Zulu sample.
